# Comprehensive in silico functional specification of mouse retina transcripts

**DOI:** 10.1186/1471-2164-6-40

**Published:** 2005-03-18

**Authors:** Samuel Shao-Min Zhang, Xuming Xu, Jinming Li, Mu-Gen Liu, Hongyu Zhao, M Bento Soares, Colin J Barnstable, Xin-Yuan Fu

**Affiliations:** 1 Departments of Pathology, Yale University School of Medicine, 310 Cedar Street, New Haven, CT 06520, USA; 2 Departments of Ophthalmology and Visual Science, Yale University School of Medicine, 330 Cedar Street, New Haven, CT 06520, USA; 3 Department of Epidemiology and Public Health, Yale University School of Medicine, 60 College Street, New Haven, CT 06520, USA; 4 Department of Genetics, Yale University School of Medicine, 333 Cedar Street, New Haven, CT 06520, USA; 5 Departments of Pediatrics, Biochemistry, Orthopaedics, Physiology and Biophysics, The University of Iowa Roy J. and Lucille A. Carver College of Medicine, 375 Newton Road, Iowa City, IA 52242, USA; 6 Department of Neurobiology, Yale University School of Medicine, 333 Cedar Street, New Haven, CT 06520, USA

## Abstract

**Background:**

The retina is a well-defined portion of the central nervous system (CNS) that has been used as a model for CNS development and function studies. The full specification of transcripts in an individual tissue or cell type, like retina, can greatly aid the understanding of the control of cell differentiation and cell function. In this study, we have integrated computational bioinformatics and microarray experimental approaches to classify the tissue specificity and developmental distribution of mouse retina transcripts.

**Results:**

We have classified a set of retina-specific genes using sequence-based screening integrated with computational and retina tissue-specific microarray approaches. 33,737 non-redundant sequences were identified as retina transcript clusters (RTCs) from more than 81,000 mouse retina ESTs. We estimate that about 19,000 to 20,000 genes might express in mouse retina from embryonic to adult stages. 39.1% of the RTCs are not covered by 60,770 RIKEN full-length cDNAs. Through comparison with 2 million mouse ESTs, spectra of neural, retinal, late-generated retinal, and photoreceptor -enriched RTCs have been generated. More than 70% of these RTCs have data from biological experiments confirming their tissue-specific expression pattern. The highest-grade retina-enriched pool covered almost all the known genes encoding proteins involved in photo-transduction.

**Conclusion:**

This study provides a comprehensive mouse retina transcript profile for further gene discovery in retina and suggests that tissue-specific transcripts contribute substantially to the whole transcriptome.

## Background

The retina is a well-defined portion of the Central Nervous System (CNS) that has long been used as a model for CNS development and function [1-4]. It is susceptible to a variety of diseases that can lead to vision loss or complete blindness. Most of the unique functions of the retina depend upon its tissue-specific transcript sets, suggesting that a systematic definition of retinal transcripts would be an invaluable approach to understanding retinal cell identities and functions.

The complete genome sequences of human and mouse provide a new starting point for understanding specific expressed transcripts, especially the sequences associated with development and disease. Expressed sequence tag (ESTs) databases, are the most abundant resource of gene expression data. Recently, Okazaki et al. used available data on ESTs to establish a comprehensive full-length transcript data base [5]. There have been a number of studies of retinal transcripts [6-10] and initial databases listing some of the retinal transcripts ([11]; ; [12]; ), but none of these have provided a global view of retinal transcripts. Recently, Blackshaw et al used serial analysis of gene expression (SAGE) identified 1,051 genes that showed developmentally dynamic expression in mouse retina [13]. Schulz and coworkers analyzed a set of retina transcripts from a mixed population of different datasets and suggested that about 13,000 transcripts might describe 90% of the adult retinome [14]. Although a systemic analysis of mouse retina ESTs has recently been reported [15,16], the functional specification of retina transcripts has not. Thus, this study provides a complementary view of mouse retina transcripts.

In the present study, we have generated mouse retina ESTs from embryonic day 13.5 (E13.5), postnatal day 1 (PN1), and adult (8 weeks old) and then analyzed about 81,000 ESTs along with other three major mouse retina libraries through an approach that integrates computational and retina tissue-specific microarray data to identify a set of candidate genes highly related to retina-specific function and retinal diseases. 33,737 non-redundant sequences were identified as retina transcript clusters (RTCs) and step by step classified into neural, retinal, late-generated retinal, and photoreceptor-enriched RTCs. This study also provides a comprehensive table of mouse retina transcript profiles that will now allow a better understanding of retinal development and function.

## Results

### Purification and specification of mouse RTCs

**Figure 1:**
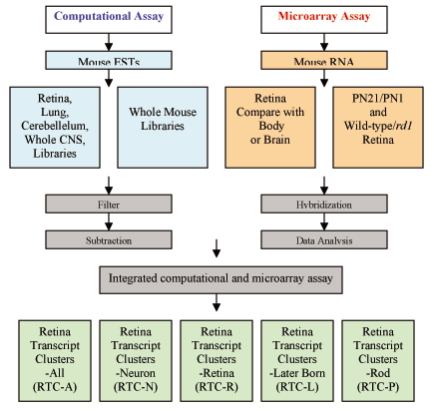
**Scheme of computational and biological procedures**. For computational analysis, five mouse EST sets were used (retina, cerebellum, lung, CNS, and whole EST). All ESTs were filtered through a non-redundant procedure (details in additional data file 1 and 2). The whole set of RTCs representing all retina transcripts was called RTC-All (RTC-A). Then RTC-A were subtracted with CNS and whole mouse ESTs to generate RTC-N and RCT-R pool. Cleaned cerebellum and lung EST pool were used as internal control. In microarray assays, tissues from PN21 mouse retina, brain, and other body regions were used for comparison of gene expression to verify RTC-R. Gene expression profiles from PN21/PN1 retina comparisons represented a set of genes involved in late-born retina development. By comparison of this set with RTC-R, RTC related to late-born retina cell development were generated (RTC-L). PN35 wild-type retina was used to compare with *rd1 *mutant retina at same age. Results from this subtraction represent a set of genes whose expression is associated with rod photoreceptors (RTC-P).

**Figure 2:**
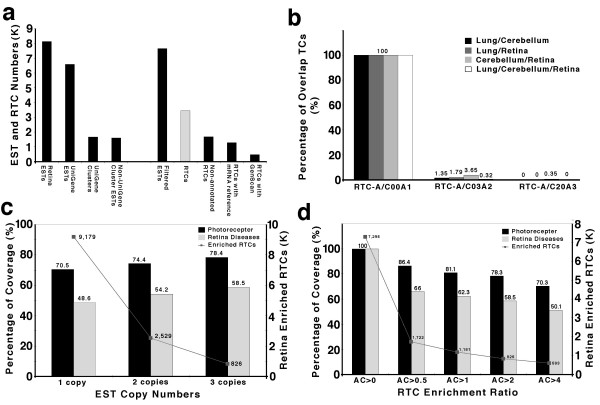
**Purification and classification of mouse retina ESTs**. (**a**) Distribution of retina ESTs in different categories. Total numbers of ESTs in mouse retina libraries; ESTs clustered by UniGene; Numbers of UniGene clusters; ESTs not clustered by UniGene; filtered ESTs for TC classification; retina transcript clusters (RTCs); Numbers of RTCs only represented in ESTs (non-annotated RTCs); Numbers of RTCs represented in mRNA references; and Numbers of RTCs represented in GenScan. (**b**) The percentage of overlap UniGene clusters between two different tissues or among three different tissues at distinct subtraction grades. (**c and d**) The coverage rates of known retina disease genes in retina libraries at different EST copy numbers and distinct subtraction grades. photoreceptor, photoreceptor related genes; Retina diseases, known retina disease genes; Enriched RTCs, retina specific and enriched RTCs.

We used a series of computational steps (Fig. 1, Additional data file 1 and 2) to clean up and reorganize a total of 81,253 mouse retina ESTs from the NCBI database (October 2002). The starting pool of retina ESTs was generated from the total retina ESTs by subtraction of a population (4,848 or 5.9% of the total) containing repeat sequences, fusion sequences, low sequence quality, vector sequences, very short sequences, mitochondrial sequences, and sequences with no BLAST-hit in the mouse genome (data not shown). 33,737 ESTs from this starting pool of 76,467 were identified by applying a program that searched for non-redundant sequences (Fig. 2A). We have termed these Retina Transcript Clusters (RTCs).

Among the RTCs, 12,939 (37.4%) have a reference RNA sequence in the NCBI database , 4,825 (13.9%) have no reference RNA sequence but do have GenScan [17] predicted sequence information, and 16,494 (47.7%) are pure EST (Fig. 2A). The whole set of RTCs was further analyzed by comparing their sequences (BLAST score > = 100) with 60,770 RIKEN mouse full-length cDNAs derived from multiple tissues [5]. 39.1% of the RTCs were not present in the RIKEN mouse full-length pool, indicating the existence of a substantial population with either unique full-length or uniquely spliced transcripts in retina.

To find criteria by which we could define tissue-enriched or tissue-specific transcript clusters we carried out a comparative analysis starting with approximately 106,000 lung and 80,000 cerebellum ESTs. These sequence pools were filtered using the same procedures as for retina to give transcript clusters (TCs) for each tissue. We then collected all the sets of mouse EST data and removed from them any set that would contain a contribution from retina, lung or cerebellum. This gave a set of data containing over 2 million ESTs. We then used this total EST dataset and the individual transcript clusters (TCs) to derive a ratio for the number of copies in each TC compared with the number in the total. Any EST in a TC that was not found in the total was set to a value of 100. An EST with 5 copies in a TC and 5 copies in the total would have a ratio of 1 and an EST with 10 copies in a TC and 5 in the total would have a ratio of 2. This analysis was carried out for each of the three TCs. We then asked at what ratio were ESTs in the RTC not found in either the LTC or the CTC using the subset of sequences for which fell into UniGene clusters. At a ratio of 0.3 and above only 1.79% of RTC were shared with lung and only 3 % with cerebellum (Fig 2B). By increasing the ratio to 2, the overlap with lung fell to 0% and with cerebellum fell to 0.3%.

We have also examined abundance in the EST pool as a criterion for helping define tissue specific transcripts. As a reference for the validity of these criteria we tested the inclusion of a known set of 47 known photoreceptor genes. Using an enrichment ratio of 2 from the above analysis, we found that 70–80% of these known photoreceptor specific genes were included in the RTCs. By increasing the copy number to 2 or more or to 3 or more, the total pool of RTCs decreased from 9,179 to 2,629 and 826 respectively without any substantial loss of detection of the known photoreceptor specific genes (Fig 2C). The pool of 826 RTCs is listed in Additional data file 3.

We also carried out this analysis by setting the copy number to > 3 and examining the effect of varying the enrichment ratios (Fig 2D). At this copy number, increasing the enrichment ratio above 0.5 only changed the coverage and total number of ESTs by small amounts. Trend tests of the reduction of RTC numbers associated with increased EST copy number were significant using photoreceptor related genes (*P *= 6.8 × 10^-59^) as criteria. Together our analysis suggests that about 80% of tissue-specific or -enriched transcripts can be identified using the two criteria, a copy ratio of at least two in the specific tissue and at least three EST copies.

### Biological approach in identification of enriched retina transcripts

**Figure 3:**
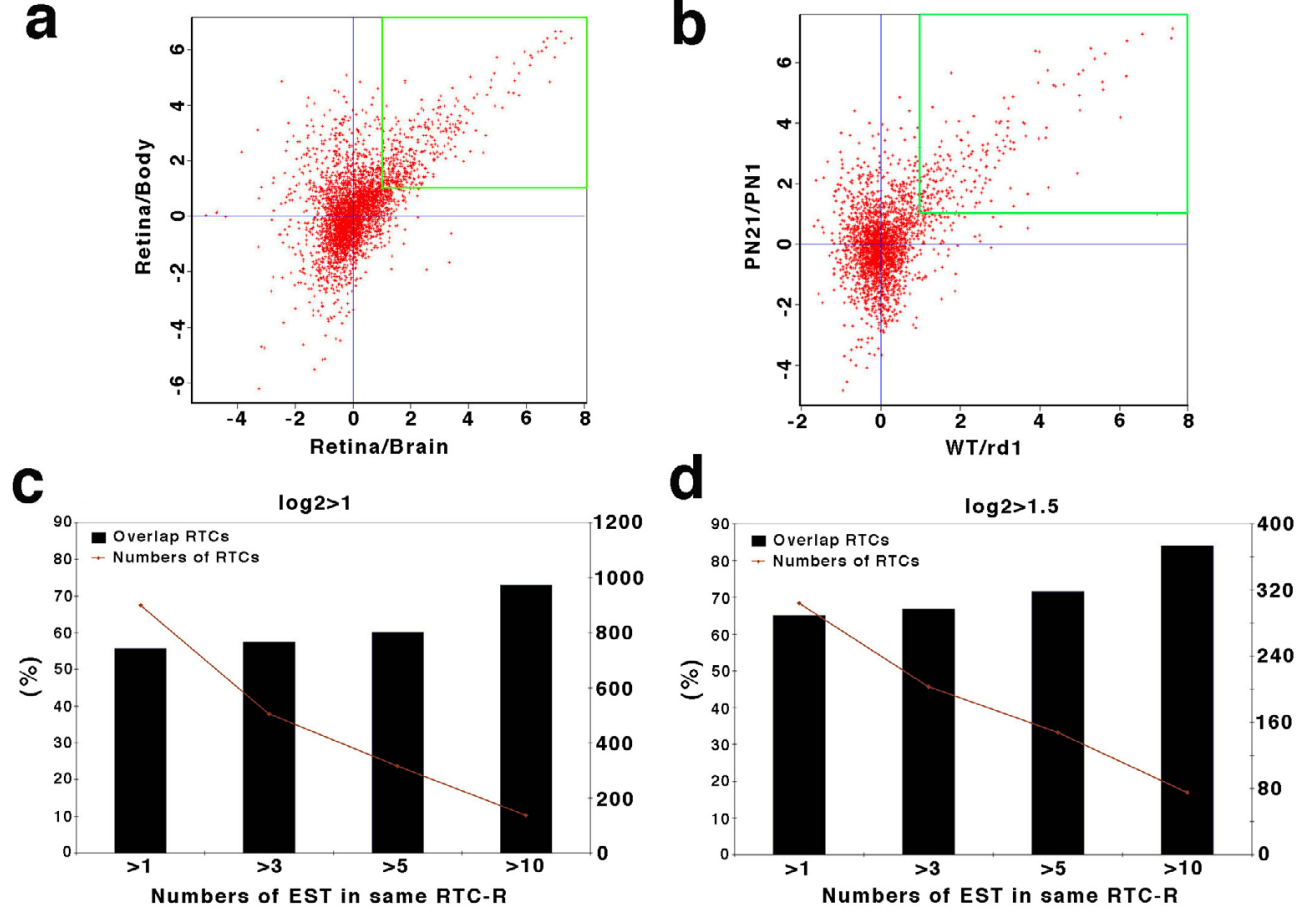
**Microarray approach and confirmation of retina enriched RTCs**. (**a**) Spot-plot graph of microarray data from Retina/Brain and Retina/Body. Log2 ratio is used for comparison. Overlap populations on upper-right were retina specific/enriched genes. (**b**) Spot-plot graph of microarray data from PN21/PN1 and WT/*rd1*. Log2 ratio is used for comparison. Overlap populations in upper-right were photoreceptor specific/enriched genes. (**c and d**) The percentage (bar-graph) of RTCs overlapping between computational assay (RTC-R) and microarray assay at distinct microarray ratio, log2 = 1 (C) and log2 = 1.5 (D). Line represents the numbers of RTCs. X-axis is copy number in same RTCs.

We also tested the biological robustness of the computational data using mouse retina tissue-specific microarrays (9,216 spots and 7,612 UniGene clusters) for experimental confirmation. Two groups of experiments were designed to detect genes enriched in retina or photoreceptors by microarray analysis. In the first group, total RNA from postnatal day 21 (PN21) retina was isolated and compared with RNA from whole brain or pooled RNA from other organs, including heart, lung, spleen, liver, and kidney from the same animals, (designated as "body" in this study). The data from Retina/Body, Retina/Brain were analyzed individually and log2 ratio results of the two experiments are shown as a spot-plot graph (Fig. 3A). The Retina/Body ratio is represented along the Y-axis and the Retina/Brain ratio along the X-axis. When setting log2 > = 1 as positive ratio, a double positive population lying at the upper-right represents retina-enriched genes. Using the same protocol, for the second group of experiments we compared RNA from PN21 retina with RNA from PN1 retina as set 1 (PN21retina/PN1retina) and PN35 wild-type retina with PN35 *rd-1 *(photoreceptor-deficient) retina as set 2 (WTretina/*rd1*retina). In set 1 we are comparing retinas before and after the generation of rod photoreceptors and in set 2 we were comparing wild type retinas with retinas in which the rods had degenerated. Thus, when setting log2 > = 1 as positive ratio, a double positive gene population lay at the upper-right and was considered as rod-enriched genes (Fig. 3B). The microarray results were used to generate two gene-expression profiles specific for later born neurons of retina and photoreceptors, respectively (Additional data file 4).

Computationally enriched ESTs from retina (RTC-R), subtracted with whole body and neuron ESTs, were compared with the microarray data from Retina/Body and Retina/Brain. The overlap between the two approaches was 70% under the microarray criterion of log2 ratio > = 1 (Fig. 3C, 316 RTCs, *P *= 6.1 × 10^-5^) and 80% using the criterion of 1.5 (Fig. 3D, 148 RTCs, *P *= 10^-4^). The percentage of overlap increased by increasing the EST copy number (Fig. 3C and 3D), indicating that the accuracy of the enriched detection in specific tissues depends on the EST copy numbers, reflective of mRNA abundance.

### Functional clusters of retina enriched RTCs

**Figure 4:**
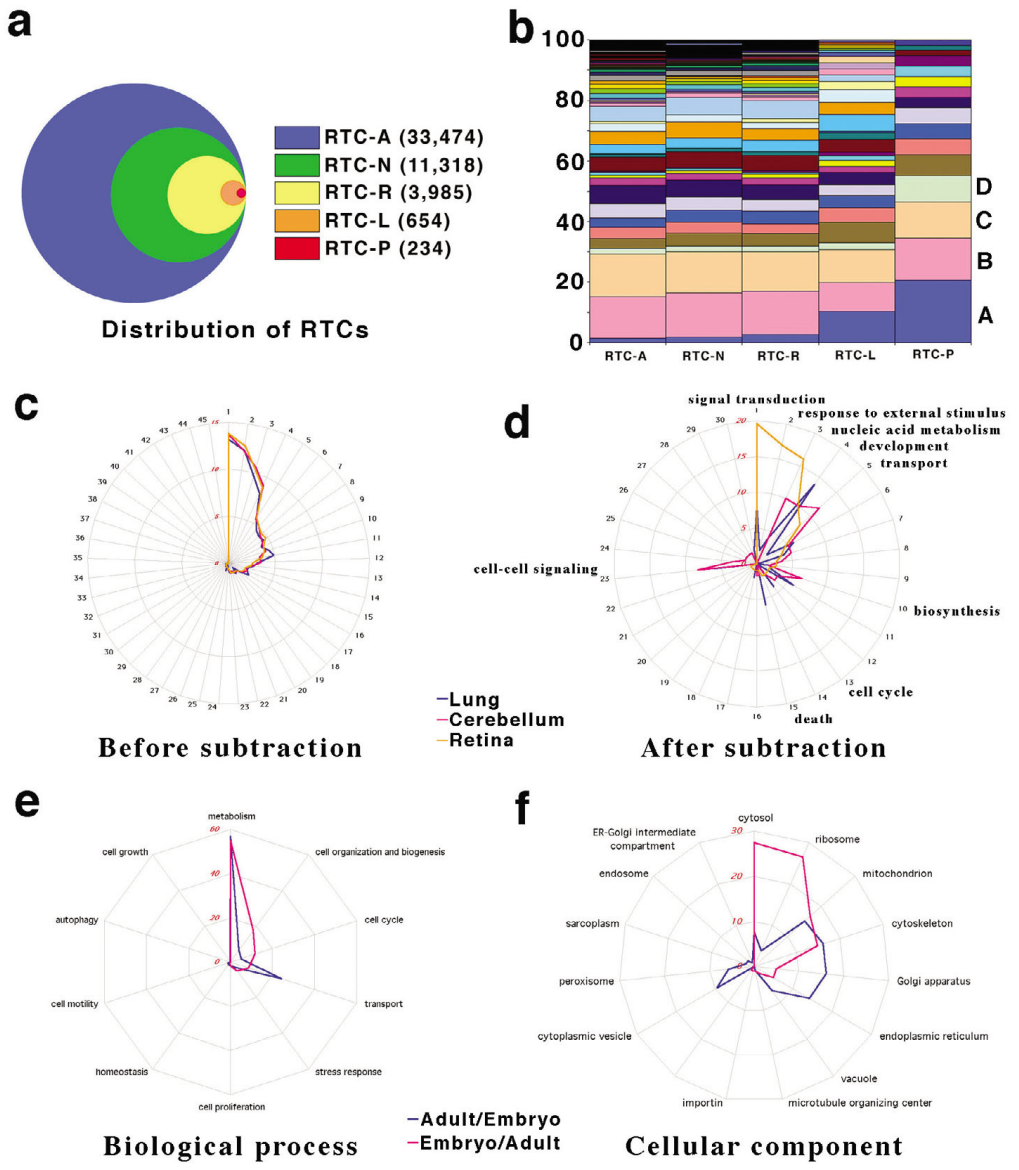
**GO analysis before and after RTC subtraction**. (**a**) Distribution of RTCs in distinct categories (details described in text). (**b**) Functional annotation in distinct categories. *A*, perception of external stimulus; *B*, nucleobase, nucleoside, nucleotide and nucleic acid metabolism; *C*, protein metabolism and modification; *D*, carbohydrate metabolism; *E*, ion transport; *F*, protein transport; *G*, organogenesis; *H*, catabolism; *I*, biosynthesis; *J*, electron transport; *K*, energy pathways; *L*, amino acid and derivative metabolism; *M*, cytoplasmic transport; *N*, phosphate metabolism; *O*, hydrogen transport. (**c and d**) Subcategories of *Biological Process *category Functional clusters of TCs in lung (blue line), cerebellum (red line), and retina (yellow line) before (C) and after (D) subtraction. (**e and f**) Functional clusters of RTCs enriched in adult (blue line) or in embryonic phase (red line). Subcategories of *Biological Process *category (E) and of *Cellular Component *category (F).

RTCs were further specified as RTC-A (RTC-all retina transcripts), RTC-N (RTC-neuron enriched transcripts), RTC-R (RTC-retina enriched transcripts), RTC-L (RTC-late-born retina cell enriched transcripts), and RTC-P (RTC-photoreceptor enriched transcripts) using the methods described in Figures 1 and additional data file 1. The number of RTCs in individual RTC categories is shown in Figure 4A. RTCs that were present in the Gene Ontology (GO) database [18] were used for functional clustering. As shown in Figure 4B within the *Biological Process *category, a significant population (10% to 20%) in the subcategory *Response to External Stimulus *is found in RTC-L and RTC-P. The majority of RTCs in this category are the genes related to light response.

To determine whether the enriched TCs of different tissues contained different functional clusters, we analyzed the EST populations of lung, cerebellum, and retina that are in the GO database. About 45 functional clusters from the *Biological Process *and *Molecular Function *categories of the GO database were analyzed before subtraction and about 30 functional clusters were analyzed after subtraction and the results shown in radar graphs. Interestingly, before subtraction, a similar distribution of functional clusters appeared among the three libraries in either *Biological Process *(Fig. 4C) or *Molecular Function *(data not shown) categories. After subtraction, using the criteria of > 3 EST copies and an enrichment ratio of 2, the distribution of functional clusters shifted in specific directions in *Biological Process *(Fig. 4D, Table 1) categories. This suggests that using subtracted subsets for classification of ESTs in individual tissues can provide accurate information about functional specificities.

**Table 1:** Comparison of Functional Clusters in Three Tissue Specific or Enriched EST Pools*

	**Retina**	**Lung**	**Cerebellum**
**GO:0007154 cell communication **(total gene numbers)	36	8	16
GO:0009605 response to external stimulus	19	1	0
GO:0009581 perception of external stimulus	18	0	0
GO:0009628 response to abiotic stimulus	18	0	0
GO:0009314 radiation response	18	0	0
GO:0009416 light response	18	0	0
GO:0009582 perception of abiotic stimulus	18	0	0
GO:0009583 perception of light	18	0	0
GO:0009591 perception of mechanical stimulus	1	0	0
GO:0009592 perception of sound	1	0	0
GO:0007600 sensory perception	17	0	0
GO:0007601 vision	17	0	0
GO:0007605 hearing	1	0	0
GO:0007609 mechanosensory perception	1	0	0
GO:0009607 response to biotic stimulus	2	0	0
GO:0007155 cell adhesion	4	2	3
GO:0016337 cell-cell adhesion	1	0	1
GO:0007165 signal transduction	22	4	9
GO:0007166 cell surface receptor linked signal transduction	10	3	4
GO:0007167 enzyme linked receptor protein signaling pathway	1	0	0
GO:0007186 G-protein coupled receptor protein signaling pathway	8	0	3
GO:0007187 G-protein signaling, coupled to cyclic nucleotide second messenger	1	0	1
GO:0008277 regulation of G-protein coupled receptor protein signaling pathway	1	0	0
GO:0007212 dopamine receptor signaling pathway	0	0	1
GO:0007214 gamma-amino butyric acid signaling pathway	0	0	1
GO:0016055 Wnt receptor signaling pathway	1	3	1
GO:0007223 frizzled-2 receptor signaling pathway	0	3	0
GO:0007242 intracellular signaling cascade	8	0	4
GO:0019932 second-messenger-mediated signaling	1	0	1
GO:0007243 protein kinase cascade	1	0	0
GO:0007264 small GTPase mediated signal transduction	1	0	0
GO:0007267 cell-cell signaling	0	3	5
GO:0019226 transmission of nerve impulse	0	0	4
GO:0007268 synaptic transmission	0	0	4
GO:0001505 neurotransmitter maintenance	0	0	1
GO:0008037 cell recognition	1	1	0

* Cell communication category is used for this table. Tissue specific or enriched EST pools are under condition of 66% concentrated in individual libraries and at least three EST copies. There are no overlap of genes among each libraries.

We also looked at distinct functional profiles at an early phase (embryo) including E13.5, E14.5, and PN1 and an adult phase, using the criteria of > 3 copies RTCs and 83% (ratio > 5) enrichment in one phase compared to the other (Fig 4E and 4F). We found, that in retina-enriched RTC-pools (826 RTCs, > 3 EST copies, 2 fold enrichment) the majority of the RTCs were found in both phases (495 RTCs, 59.9%). 34.4% (284 RTCs) were found only in the adult phase, and 5.7% (47 RTCs) were found only in the embryo phase.

To examine the different functional clusters in these two phases, categories of the GO database were analyzed. In the biological process category, 18% of RTCs belonged to *Cell Organization and Biogenesis *clusters and 12% to *Cell Cycle *clusters in the embryo phase compared with 6% and 5%, respectively, in the adult phase. Conversely, about 24% of RTCs were in the *Transport *clusters in the adult phase and only 8.5% in the embryo phase (Fig. 4E). Although there was the same percentage in *Metabolism *clusters (Fig. 4E) in both the embryo (55%) and adult (56%) phases, detailed analysis showed that about 17% of RTCs were in *Biosynthesis *clusters in the embryo phase compared with 7.7% in the adult phase (Fig. 4F). Interestingly, *Lipid metabolism *(5.8%) and *Energy pathways *(3.9%) were significantly higher in adult phase and in embryo phase, respectively. As expected, overall comparison of these phases shows a change from functions characteristic of a proliferating epithelium (such as cell cycle) to those characteristic of a mature retina (such as transport).

### A comprehensive transcript profile of mouse retina

The computational processes described above classified a total of 33,737 RTCs for their statistical probability of being retina specific or retina-enriched, as listed in Additional data file 5. Because of the possibility of alternative splicing, we did not combine all UniGene clusters together and kept the non-redundant sequences as our cluster units. Thus, there may be some gene redundancy in the 33,737 RTCs. If we ignore this potential redundancy among the RTCs, 25,673 RTCs can be clustered into 14,618 UniGene clusters (57%) and 8,064 RTCs have not yet been clustered. If we assume the same ratio of RTCs to clusters for un-clustered RTCs, we estimate that about 19,000 to 20,000 genes might expressed in mouse retina from embryonic to adult stages.

Seven sets of information are provided in the additional data file 5. First, a set of basic information including RTC I.D. numbers, Genbank I.D. for reference sequences, locus link numbers, UniGene numbers and descriptions; second, chromosomal locations in the mouse (UCSC, mm2, Feb 2002) and human (UCSC, hg13, Nov, 2002) genomes including start and end points within the UCSC golden-path database; third, information about TC numbers in whole mouse ESTs, whole mouse retina libraries, adult retina, and embryonic retina libraries; fourth, ratio of RTC enrichment compared with whole mouse ESTs, whole ESTs from neuronal libraries, and also a comparison between RTC from adult and embryonic libraries; fifth, RTC enriched patterns under different enrichment criteria; sixth, published SAGE information from Blackshaw et al [6], and seventh, human retina transcript information from RetBase [7].

**Figure 5:**
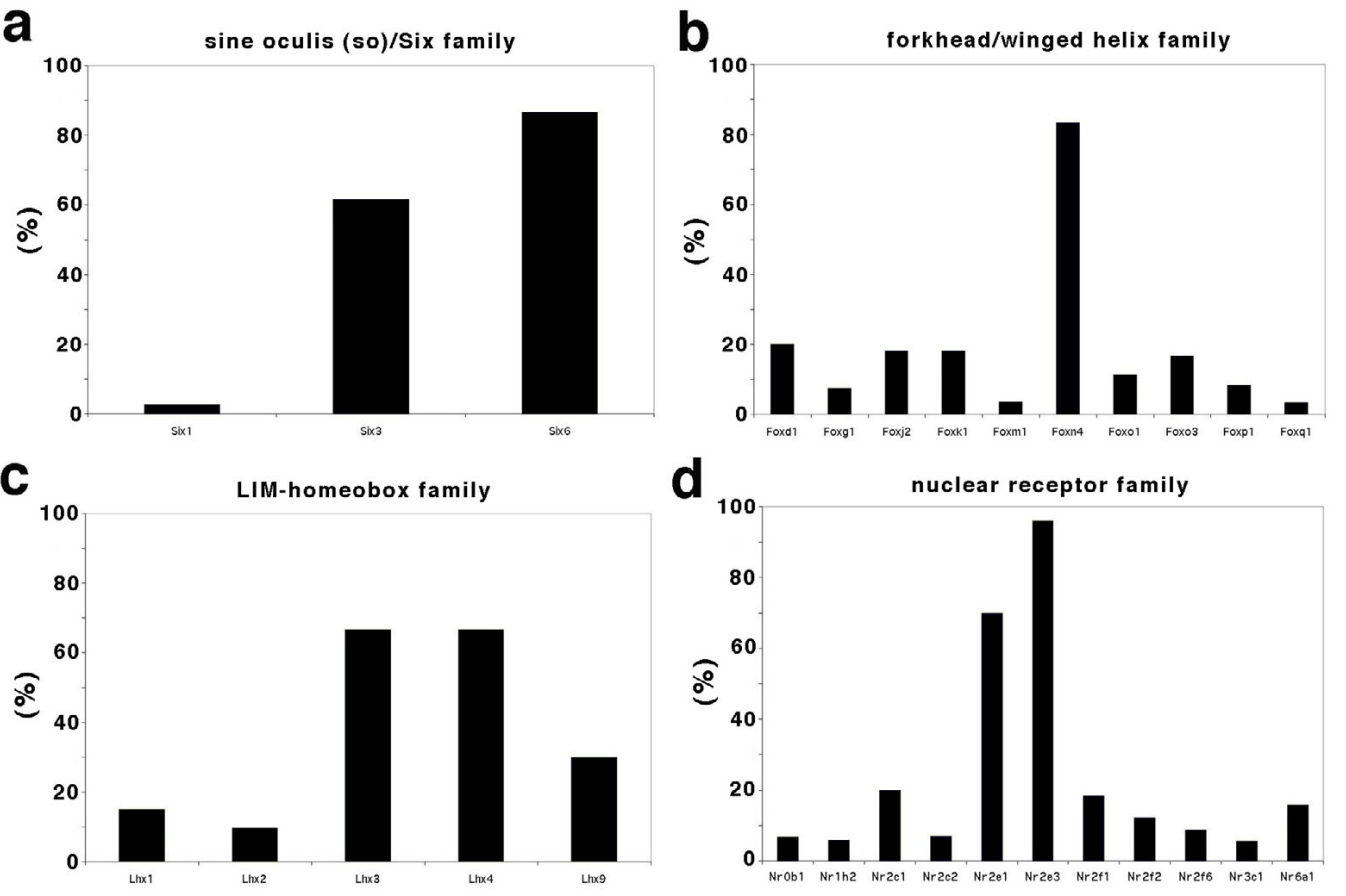
**Distribution of enriched RTCs in gene families**. (**a**) The percentage of enriched RTCs of *sine oculis (so)*/Six family in retina. (**b**) The percentage of enriched RTCs of forkhead/winged helix family in retina. (**c**) The percentage of enriched RTCs of LIM-homeobox family in retina. (**d**) The percentage of enriched RTCs of nuclear receptor family in retina.

Using the RTC database, we can identify new genes specifically expressed or enriched in the retina for which there is not yet any biological evidence. For example, a total of 37 known genes with homeodomains from the RTC pool are listed in Table 2. Some of the genes have been well studied like Crx [19,20], Rax [21], Otx2 [22,23], and Prox1 [24]. Most of these genes, however, have not previously been described in retina, such as Og9x [25], Lhx [26,27], and Onecut [28,29]. Three of the *sine oculis (so)*/Six family of genes were present in the RTC pool (Fig. 5A). ESTs of Six3 and Six6 were 60% to 80% enriched in retina. The patterns have been confirmed by biological studies of expression level [30] and function analysis [31-33] of these genes. Ten members of forkhead/winged helix family appeared in our RNA pools and only one, Foxn4 is highly concentrated in the retina (Fig. 5B). This observation has also been confirmed by a recent study [34]. The genes from those two gene families are highly enriched in the embryo phase. Lhx3 and Lhx4, from the LIM homeodomain gene family, are highly enriched in the adult retina (Fig. 5C). Similarly, genes such as those encoding members of the guanine nucleotide binding protein family, the ATP-binding cassette protein family, the voltage-dependent calcium channel protein family, and the potassium voltage-gated channel protein family (additional data file 6) are also present in the retina enriched pool. We do not have biological evidence for all the genes that have been listed in this comprehensive profile of RNAs expressed in retina, yet our gene-tables can be useful tools for analyzing mouse retina transcripts and can provide an overview of genes involved in function and development of retina.

**Table 2:** Information of Known Homeodomain Contained Genes from RTCs

**Symbl**	**%**	**D/E (%)**	**E/D (%)**	**A**	**C**	**RTCs**	**UniGene**	**Gene Name**	**MmChr**	**HsChr**	**Ref**
Crx	98.7	99.9	0.1	74	1	BU503524	Mm.8008	cone-rod homeobox containing gene	chr7	chr19	y
Dlx1	50	0	100	1	2	BG808909	Mm.4543	distal-less homeobox 1	chr2	chr2	y
Dlx2	100	0	100	1	0	BG805973	Mm.3896	distal-less homeobox 2	chr2	chr2	n
Hhex	5.6	100	0	1	17	BB709075	Mm.33896	hematopoietically expressed homeobox	chr19	chr10	n
Hmx1	100	0	100	3	0	BE949806	Mm.10104	H6 homeo box 1	chr5	chr4	y
Hoxc4	5.9	100	0	1	16	BB283935	Mm.1351	homeo box C4	chr15	chr12	n
Hoxc8	20	100	0	1	4	BB283726	Mm.6167	homeo box C8	chr15	chr12	n
Irx2	1.4	0	100	2	139	BG801773	Mm.28888	Iroquois related homeobox 2 (Drosophila)	chr13	chr5	n
Irx3	5.7	0	100	2	33	BE951617	Mm.39039	Iroquois related homeobox 3 (Drosophila)	chr8	chr16	n
Irx5	6.8	0	100	5	69	BE949849	Mm.101153	Iroquois related homeobox 5 (Drosophila)	chr8		y
Irx6	100	100	0	1	0	BG298876	Mm.137247	Iroquois related homeobox 6 (Drosophila)	chr8	chr16	y
Isl1	20.6	38.5	61.5	14	68	BF467775	Mm.42242	ISL1 transcription factor, LIM/homeodomain, (islet-1)	chr13	chr5	n
Lhx1	17.1	100	0	3	17	BB283776	Mm.4965	LIM homeobox protein 1	chr11	chr17	n
Lhx2	9.7	33.3	67.7	7	65	BF462761	Mm.142856	LIM homeobox protein 2	chr2	chr9	y
Lhx3	66.7	100	0	12	6	BE986454	Mm.15655	LIM homeobox protein 3	chr2	chr9	n
Lhx4	66.7	100	0	2	1	BG297508	Mm.103624	LIM homeobox protein 4	chr1	chr1	n
Lhx9	30	0	0	3	7	BE982177	Mm.79380	LIM homeobox protein 9	chr1	chr1	n
Nkx6-2	3.8	67.7	33.3	7	175	BE949669	Mm.28308	NK6 transcription factor related, locus 2 (Drosophila)	chr7	chr10	n
Og9x	80	100	0	4	1	BI736847	Mm.142724	OG9 homeobox gene	chr11		n
Onecut1	33.3	0	100	1	2	BG805378	Mm.3512	one cut domain, family member 1	chr9	chr15	n
Onecut3	100	0	0	1	0	BE995314	Mm.221027	one cut domain, family member 3	chr10	chr19	n
Otx2	42.9	100	0	9	12	BG404413	Mm.134516	orthodenticle homolog 2 (Drosophila)	chr14	chr14	y
Pax6	44.2	65.4	34.6	46	49	BQ930162	Mm.3608	paired box gene 6	chr2	chr11	y
Pknox1	4.5	0	0	1	21	BM941536	Mm.87619	Pbx/knotted 1 homeobox	chr17	chr21	n
Pknox2	37.5	0	100	3	5	BG803156	Mm.41577	Pbx/knotted 1 homeobox 2	chr9	chr11	n
Prox1	60	100	0	6	4	BM940687	Mm.20429	prospero-related homeobox 1	chr1	chr1	y
Prrx1	14.3	100	0	2	12	BB283141	Mm.3869	paired related homeobox 1	chr1	chr1	n
Rax	100	66.7	33.3	12	0	BB642844	Mm.3499	retina and anterior neural fold homeobox	chr18	chr18	y
Six1	2.6	100	0	1	37	BB283914	Mm.4645	sine oculis-related homeobox 1 homolog (Drosophila)	chr12	chr14	n
Six3	76.9	0	100	10	3	BG807874	Mm.15630	sine oculis-related homeobox 3 homolog (Drosophila)	chr17	chr2	y
Six6	86.7	42.9	57.1	13	2	BI990712	Mm.57138	sine oculis-related homeobox 6 homolog (Drosophila)	chr12	chr14	y
Vax2	50	0	100	1	1	BI989827	Mm.57253	ventral anterior homeobox containing gene 2	chr6	chr2	y
Vsx1	100	100	0	4	0	BB642331	Mm.207061	visual system homeobox 1 homolog (zebrafish)	chr2		y
Zfh4	100	100	0	3	0	BB642530	Mm.41522	zinc finger homeodomain 4	chr3	chr8	n
Zfhx1a	10.1	42.9	57.1	8	71	BE954320	Mm.3929	zinc finger homeobox 1a	chr18	chr10	n
Zfhx1b	10.7	100	0	3	25	BI730214	Mm.37676	zinc finger homeobox 1b	chr2	chr2	n
Zhx1	75	100	0	3	1	BG404047	Mm.37216	zinc fingers and homeoboxes protein 1	chr15	chr8	n

%, percentage of RTCs distributed in retina

A, RTCs; C, filtered mouse ESTs

D/E (%), percetage of RTCs in adult phase; E/D (%), percetage of RTCs in embryonic phase

MmChr, mouse chromosome; HsChr, human chromosome

Ref, genes have been study in retina (y) or not (n)

From the RTC database, we can also extract identify candidate genes for human retinal diseases. As shown in Figure 2C, using the criteria of enrichment ratio > 2 and at least 3 EST copies in RTC, 826 RTCs were identified as a retina enriched gene pool. By homology with the human genome, this RTC pool covers about 80% of known-photoreceptor related genes (29 out of 37, Fig. 2C and 2D). Through comparison with individual interval gene numbers, numbers of candidate RTCs are concentrated to 1.5% to 0.05% (additional data file 7). A full list of known human retina disease genes and the subtraction information are shown in additional data file 8. Among retina-disease-related genes, almost all photoreceptor related genes especially the genes involved in phototransduction processes are covered by our most stringent subtraction and selection criteria (> 3 EST copies and 2 fold enrichment). However, genes related to systemic diseases or syndromes, genes involved in RNA processing and genes with lower copy numbers in RTCs are not present under these criteria, indicating a limitation of this method. Although candidate genes from the 826 RTCs pool can cover over 41 of 48 mapped human retina disease loci, here we provide only the genes for 27 loci that have been more stringently associated with primarily photoreceptor disorders (additional data file 7 and 9).

Comparison with other approaches for retina enriched gene subtraction shows obvious gaps among the various experimental approaches (Table 3). Here we have compared our enriched gene pool with the pools from SAGE [6] and RetBase [7]. Only genes clustered by UniGene are used using two criteria from SAGE pool with two standards [6], 2 out of 4 (71 genes) and SAGE 264 (264 genes) respectively; one criteria from Retbase (373 genes, [7]); and one criteria with at least 3 EST copies and 2 fold enrichment in RTCs (184 genes). The highest 22% overlaps between our and SAGE pools and the lowest 5% overlaps between Retbase and SAGE pools.

**Table 3:** Comparison of predicted retina enriched genes by different system

	Sage71	Sage264	RetBase374	RTC184
Sage71	**71(100)**	29(40.8) 5(7)	22(30.9)	
Sage264	29(10.9)	**264(100)**	4 (1.5)	15(5.7)
Retbase374	5(1.3)	4(1.1)	**373(100)**	14(3.7)
RTC184	22(11.9)	15(8.2)	14(7.6)	**184(100)**

Sage 71, 2 out of 4 (Blackshow et al, 2001)

Sage264, (Blackshow et al, 2001)

RetBase374, (Katsanis et al. 2002)

## Discussion

The full specification of transcripts in an individual tissue or cell type can greatly aid the understanding of the control of cell differentiation and cell function. In the present study, we have integrated computational bioinformatics and microarray experimental approaches to classify the tissue specificity and developmental distribution of mouse retina transcripts.

We have defined 33,737 retina transcript clusters (RTCs) as single units with non-redundant sequences, although multiple transcript clusters can be in the same UniGene cluster. Such RTCs may represent different parts of a gene, or splice variants that are not considered in the UniGene database. We have calculated that about 19,000 to 20,000 genes may be expressed during mouse retina development from embryonic day 13.5 to adult. This is about 30% more compared with Schulz's Retinome (13,037 genes) [14], although this was restricted to transcripts in the adult phase. Since the nervous system, including the retina, has more prolific RNA splicing (2.5 fold higher that other tissues) [35], we suggest that the numbers of unique transcripts found in retina may well be greater than 20,000. More interestingly, since 39.1% of the RTCs are not included in the most comprehensive mouse full-length transcript set made by RIKEN [5], tissue-specific transcripts and splicing may constitute a substantial proportion of the whole transcriptome. On the other hand, there are large numbers of ESTs (about 16,000) that do not appear to encode proteins. Many of these may serve as regulatory RNA or have other unknown functions and need further study [5,13].

A major concern for specification of transcripts from ESTs is how to confirm a reliable result. We have used four control steps to verify the results. First, as an external negative control we compared the specificity in our target tissue, retina, and in other tissues (lung and cerebellum). Second, we used an internal positive control. We used known photoreceptor-specific genes to check their coverage rate during different steps of retina specification. The third control was to identify functional clusters through the Gene Ontology (GO) database before and after specification of ESTs in different tissues. Biological confirmation was the final control experiment in this study and used a mouse retina tissue-specific microarray. Results from all the control experiments fully support the conclusions of this study.

Identification of retina disease genes is an important and immediate use for genome-wide study tools. Several approaches have been used for genome-wide hunting of such genes. However, as shown in our results, the data sets show obvious gaps among these various experimental approaches (Table 3). Katsanis et al performed subtraction against 1.4 million human ESTs with 40,000 human retinal ESTs by a series of computational tools. They found a total of 925 ESTs likely to be specifically or preferentially expressed in the retina [7]. We found a low overlap between RetBase and our enriched pools. In part this is because of the smaller starting sample, the majority of human ESTs are generated from tumors and the emphasis on single copy sequences in the RetBase set. A SAGE analysis identified 264 uncharacterized genes that were specific to or highly enriched in rods [6-9]. This data set showed more overlap with our results but there are, still obvious gaps among these various experimental approaches, so we suggest that integrating different approaches might be much more valuable for tissue specific and enriched gene prediction.

Generation and specification of an entire transcription profile for individual tissues or cells with specific functions and morphologic identities represents the next major task in the genome era. In conclusion, this study complements and extends previous studies in a number of ways. First, we have generated a comprehensive data set of retina transcript profiles with functional and developmental explanation, as the examples shown in Figure 4 and Table 2, diverse function information can be generated through our RTC profiles that will now allow a better understanding of retinal development and function. It is not only for hunting retina disease genes, but also for understanding of gene developmental distribution. Second, this study has classified retina transcripts into different grades of retinal specificities that will help us define them as common, neural specific or retinal specific genes. Third, the distinct approaches of this study will allow an easy updating of our mouse retina transcripts databases in future.

## Methods

### EST resources and manipulation

Mouse retina ESTs were collected from NCBI and were filtered and cleaned up by a series of programs (Figures 1, S1 and S2). Two Dell Precision WorkStation 530's running RedHat 7.0 Linux were used for computational processes. The database used was MySQL and the languages for programming were Python, GNU C, awk, and bash. Repeat detection used RepeatMasker  (kindly provided by A. Smith and P. Green). Other analyses used custom programs.

### Mice and retina sample collection

Mice were purchased from the Jackson Laboratory (C57Bl/6j) or were a gift from Dr. C. Zeiss, Yale University (C3H *wt *and *rd1*). Retinas were dissected without contamination from lens, iris, cornea, and ciliary body. 10 to 20 retinas or other organs were pooled for RNA isolation. All animal experiments were conducted in accordance with NIH guidelines and were approved by the IACUC of Yale University School of Medicine.

### RNA preparation

Total RNA was isolated by TRIzol (Invitrogen) and purified by RNeasy mini kit (QIAGEN). 5 μg total RNA with 280/260 ratios greater than 1.9 was used for array hybridization without amplification. Three to four sets of RNA were prepared from each age of retina and processed individually for microarray analysis.

### Microarray experiments

About 12,000 non-redundant mouse retina ESTs were generated from about 28,000 ESTs that generated from E13.5, PN1 and adult (The NIH-University of Iowa Brain Molecular Anatomy Project). 9,216 purified PCR-amplified inserts were printed by the Yale Keck Microarray Core on poly L-Lysine (Sigma) coated glass slides utilizing a GeneMachines Omnigrid robotic arrayer (GeneMachines). 3DNA Submicro EX Expression Array Detection Kits (Genisphere, PA) were used for RNA labeling. Detailed microarray experimental protocols are shown in supplemental methods. Slides were scanned on a GenePix 4000B scanner and the data were manipulated with GenePix software Version 4.0 (Axon Instruments). Three or four sets of microarray data for each experiment were used for Student t test and gene collection. Gene collection methods are described in text and also please see additional data file 10 for methods detail.

### Statistics

To test whether the features illustrated in Figures 2 and 3 show an increasing trend in terms of covering photoreceptor related genes and retina disease genes through more stringent filtering criteria, we conducted trend tests in the following form: , where *C *is the number of classes above the baseline total population, *w*_*i *_is the weight for the *i*th class, and *y*_*i *_is the observed percentage related to the feature of interest in the *i*th class. The value of *C *= 2, 4, 3, and 3, for figures 2C, 2D, 3C, and 3D, respectively. The value of *y*_*i *_is the proportion of the ESTs with a given feature in the *i*th class, with the feature being either a photoreceptor related gene or a retina disease gene. The weights are 1, 2, ..., C for the C classes.

To assess the statistical evidence of a trend in the data based on *T*, we calculated the mean and variance of *T *under the null hypothesis of no trend conditional on the feature distribution in the baseline total population. It can be shown that when the feature of interest is binary, i.e. a given EST either has or does not have this feature,



where



*N*_*i *_is the number of ESTs in the *i*th class, *N*_0 _is the number of ESTs in the total population, and *y*_0 _is the proportion of ESTs having a given feature in the total population. The statistical significance of the observed increasing trend is



and the statistical significance of the observed decreasing trend is



where Φ is the cumulative function of the standard normal distribution. Statistic calculations were done by R 1.8.1 or R 1.7.1. 

## List of abbreviations

RTC, Retina Transcript Cluster; CNS, Central Nervous System; EST, Expressed Sequence Tag; GO, Gene Ontology; PN, Postnatal

## Authors' contributions

SSMZ was primarily responsible for the design, coordination, conduct, and all experiments of the studies. XYF and CJB were responsible for coordination of the studies. XX and JL were responsible for computational data analyses and software development. SSMZ and MGL were responsible for microarray experiments and analysis. MGL and HZ were responsible for statistical analysis. SSMZ, MBS, and XYF were responsible for RNA collection and original initiation and generation of mouse retina ESTs. SSMZ and CJB drafted the manuscript and figures. All authors read and approved the final manuscript.

## Supplementary Material

Additional File 1 Methods for in silico purification of ESTs. Click here for file

Additional File 2 Methods for in silico specification of RTCs. Click here for file

Additional File 3 RTC list under criteria of RTC-A/C20A3. Click here for file

Additional File 4 Gene list of RTC-L and RTC-P. Click here for file

Additional File 5 Whole RTC information and SAGE, RetBase data comparison. Click here for file

Additional File 6 Samples of the percentage of RTCs in different gene families. **a**, Heterotrimeric guanine nucleotide-biding proteins. **b**, ATP-binding cassette (ABC) transporter superfamily. **c**, Voltage-dependent calcium channel proteins. **d**, Voltage-gated potassium channel proteins. Click here for file

Additional File 7 Candidate genes for human retina disease loci. **a**, Gene numbers of chromosome interval and concentrated retina enriched gene pool in human known retina disease gene loci. **b**, Concentrated ratio between retina enriched gene pool and whole interval genes of the loci for human known retina disease gene. **c**, Gene numbers of chromosome interval and concentrated retina enriched gene pool in some human known retina disease loci. **d**, Concentrated ratio between retina enriched gene pool and whole interval genes of the loci for human retina disease. Click here for file

Additional File 8 Known human retina disease gene list used in this study. Click here for file

Additional File 9 Recommended gene candidates for human retina disease loci. Click here for file

Additional File 10 Additional methods. Click here for file
