# Supplementary material for: Comprehensive in silico functional specification of mouse retina transcripts
Source: BMC Genomics. 2005 Mar 18;6:40. doi: 10.1186/1471-2164-6-40 (PMC1083414; doi:10.1186/1471-2164-6-40)
Supplement: Additional File 1 — Methods for in silico purification of ESTs. [file 1471-2164-6-40-S1.doc]

# Procedure for EST cleaning and clustering

1. get EST libraries

1.1. get list of all Mouse EST libraries, from "http://www.ncbi.nlm.nih.gov/UniGene/lbrowse.cgi?ORG=Mm&DISPLAY=ALL"

1.2. select EST libraries as set A, and set B

1.3. download sequence file of each EST library, from "http://www.ncbi.nlm.nih.gov/UniGene/download.cgi?"

2. clean bad sequences

2.1. filter bad quality sequences

2.1.1. filter EST whose high quality length is less than 50 bps

2.1.2. filter EST whose length is less than 100 bps

2.2. filter repeated sequences

2.2.1. RepeatMasker all EST

2.2.2. select EST who has over 50% repeated region

2.3. filter Mitochondrial

2.3.1. get Mouse Mitochondrial genome, from "ftp://ftp.ncbi.nih.gov/blast/db/mito.nt"

2.3.2. BLASTN all EST against Mouse Mitochondrial genome

2.3.3. RepeatMasker Mitochondrial sequence

2.3.3. select EST well matched with Mitochondrial

2.4. filter vector sequence

2.4.1. BLASTN all ESTs against vector FASTA file, from "ftp://ftp.ncbi.nih.gov/blast/db/vector.Z"

2.4.2. select BLASTN records with high simlirity and high score

2.4.3. select EST sequence containing vector sequence

2.5. filter fusion sequence

2.4.1. BLATN all ESTs against Mouse Genome, from "http://genome.ucsc.edu/cgi-bin/hgBlat"

2.4.2. select good BLAT records

2.4.3. select BLAT records caused by mRNA fusion, get fusion EST

3. cluster cleaned EST

3.1. cluster ESTs, get core EST for each cluster

3.1.1. BLASTN set A vs. set A

3.1.2. get relationship between EST with BLAST score greater than 150

3.1.3. group all ESTs with relationship greater than 150 into one cluster, get one coreEST for each cluster

3.1.4. get relationship between EST with BLAST score greater than 80

3.1.5. cluster single EST with relationship of greater than 80

3.2. cluster coreEST according to mRNA

3.2.1. select presentative mRNA of each Unigene cluster,

3.2.2. BLASTN coreEST vs. mRNA

3.2.3. for each coreEST, select BLASTN record with maxscore

3.2.4. Do RepeatMasker on mRNA

3.2.5. get relationship between coreEST and mRNA

3.2.6. cluster coreEST according relationships

3.3. cluster coreEST according to genscan predicted genes

3.3.1. get FASTA file for each genscan predicted gene,

3.3.2. select coreESTs which has no match with mRNA, as "NomRNA_coreEST"

3.3.3. BLASTN "NomRNA_coreEST" vs. genscan FASTA file

3.3.4. Do RepeatMasker on genscan FASTA file

3.3.5. get relationship between NomRNA_coreEST and genscan gene

3.3.6. cluster NomRNA_coreEST according to relationship
