# Supplementary material for: Comprehensive in silico functional specification of mouse retina transcripts
Source: BMC Genomics. 2005 Mar 18;6:40. doi: 10.1186/1471-2164-6-40 (PMC1083414; doi:10.1186/1471-2164-6-40)
Supplement: Additional File 2 — Methods for in silico specification of RTCs. [file 1471-2164-6-40-S2.doc]

**Procedures for Computational Programing**

1. Computational Environment and Tools

1.1 Computer: Dell Precision WorkStation 530 with 1.8G CPU with 512K cache, 3.0GB PC800 ECC RDRAM.

1.2 OS: RedHat 7.0 Linux system,

1.3 Language: Python, GNU C, awk, bash

1.4 DataBase: MySQL

1.5 Tools: BLAST : "ftp://ftp.ncbi.nih.gov/blast/executables/ blast.linux.tar.Z"

BLAT : "http://genome.ucsc.edu/cgi-bin/hgBlat"

RepeatMasker : provided by Dr. Arian Smith at

2. Annotation

2.1 Annotate by Unigene, Unigene data from: ftp://ftp.ncbi.nih.gov/repository/UniGene/Mm.seq.uniq.gz

2.2 Annotate by LocusLink, LocusLink data from: ftp://ftp.ncbi.nih.gov/refseq/LocusLink

2.3 Map all EST sequences to Mouse Genome, by BLAT: "http://genome.ucsc.edu/cgi-bin/hgBlat"

2.4 Get Homology Human Genome Locus for each EST_cluster:

2.4.1 if EST_cluster match with one mRNA, then BLAT this mRNA to Human Genome, get homology Human Genome Locus

2.4.2 otherwise, if EST_cluster match with one genscan sequence, then BLAT this genscan to Human Genome, get homology Locus

2.4.3 otherwise, cut Mouse genome sequence within interval (EST_Locus0 - 3k, EST_Locus1 + 3k ), do BLAT this expanded genome sequence versus Human Genome, get homology Locus.

2.5 programs:

convert_gb_ug.py : get Unigene code for each GenBank accession number in list

get_LocusL_data.py : abstract data from file "LL_tmpl" and make up a data table with strict format

UCSC_Mm_BLAT.py : map all cDNA sequences onto Mouse genome, using BLAT website.

UCSC_Hs_BLAT.py : map all cDNA sequences onto Homan genome, using BLAT website.

3. GO function cluster:

GO data from: http://www.godatabase.org/dev/database/archive/latest/go_200301-termdb-tables.tar.gz

programs :

GOcluster.py : given a list of genes with GO annotation, and several GO code,

for each GO code, search all related gene whose one or more GO annotation related with this GO code.

GOTreeCluster.py : given a list of genes with GO annotation, and depth of seaching the GO-graph tree,

for each GO node in the top part of tree within depth less than given value, search all related gene.

GO_patternmatch.py : given a list of genes with GO annotation, and one GO code,

search all genes whose one or more GO annotation is related with that GO code.

4. cDNA Microarray validation:

4.1 select media value of F635, B635, F532, B532 of each spot as the measurement value.

4.2 normalize all measurement value.

4.2.1 collect media F635 of all spots into array , then get media value of this array, as array_media_F635.

4.2.2 collect media F532 of all spots into array , then get media value of this array, as array_media_F532.

4.2.3 for each spot, normalized_F635 = media_F635 / array_media_F635

4.2.4 for each spot, normalized_F532 = media_F532 / array_media_F532

4.3 get rato for each spot: rato = log2( normalized_F635 / normalized_F532 )

4.4 process data by above method for each array experiment: Retina_Brain, Retina_Body, NormAdult_KOAdult, PN21_PN1

- 1. programs :

normalize.py : work as described in 4.1, 4.2, 4.3

5. RetBase comparision:

5.1 download accession number from : "http://www.hgsc.bcm.tmc.edu/retbase/"

5.2 download sequence from : "http://www.ncbi.nlm.nih.gov/entrez/batchentrez.cgi"

5.3 try to get homology RetBase sequence for each Retina mRNA-cluster by method based on Unigene sequence

5.3.1 download accession number from : "http://www.hgsc.bcm.tmc.edu/retbase/"

5.3.2 download sequence from : "http://www.ncbi.nlm.nih.gov/entrez/batchentrez.cgi"

5.3.3 get Unigene number for all RetBase sequence

5.3.4 get Unigene representive sequence for all Unigene number

5.3.5 BLASTN these representive sequences versus Mouse Unigene representive sequences

5.3.6 for each RetBase sequence, select uniq matched Mouse Unigene based on BLASTN score.

5.4 try to get homology RetBase sequence for each Retina mRNA-cluster by method based on Genome Locus

5.4.1 download RetBase sequence as 5.1

5.4.2 get Human Genome Locus (HsLocus_0, HsLocus_1) for each RetBase sequence by BLAT program

5.4.3 for each Retina mRNA-cluster, say A,

if its homology Human Genome Locus overlap with expanded Locus of one RetBase sequence, say B,

then take A and B as homology sequence. (expand 50 kb at both ends)

5.5 for each Retina mRNA-cluster, if there is good homology RetBase by Unigene method, take it as homology RetBase gene

otherwise, if there is good homology RetBase sequence by Genome Locus, take it as homology RetBase gene.

5.6 programs :

compare.RetBase.HsLocus.py: work as described in 5.4

RetBase.MmHomolog.py: work as described in 5.5

6. SAGE comparison:

6.1 download data from <http://www.cell.com/cgi/content/full/107/5/579/DC1>

6.2 programs:

do_sage.py : convert text data file into data table with strict format

mark_up.py : mark Unigene code which have multi-tag, and Sage tag which occur in multi-UniGene

7. Human retina disease genes:

| **Name** | **Chromosome** | **Human Ref** | **Mouse Ref** | **Core RTCs** |
| --- | --- | --- | --- | --- |
| GNAT2 | 01p13.1 | NM_005272 | NM_008141 | BU505641 |
| ABCA4 | 01p21-p22 | NM_000350 | NM_007378 | BU504330 |
| RPE65 | 01p31 | NM_000329 | AF410461 | BM932603 |
| CRB1(RP12) | 01q31-q32.1 | NM_012076 | NM_133239 | BU506277 |
| ALMS1 | 02p13 | NM_015120 | NM_145223 | BE981177 |
| EFEMP1 | 02p16-p21 | NM_004105 | BC023060 | BI730321 |
| CNGA3 | 02q11 | NM_001298 | AJ243933 | BI730059 |
| SAG | 02q37.1 | NM_000541 | NM_009118 | BQ921528 |
| GNAT1 | 03p22 | NM_000172 | NM_008140 | BU505975 |
| RHO (RP4) | 03q21-q24 | NM_000539 | BC013125 | BU506109 |
| USH3A | 03q21-q25 | NM_052995 | NM_052995 | BU505290 |
| OPA1 | 03q28-q29 | NM_015560 | AB044138 | BB642686 |
| PROML1 | 04p | NM_006017 | NM_008935 | BU505217 |
| CNGA1 | 04p12-cen | NM_000087 | U19717 | BQ946527 |
| WFS1 | 04p16.1 | NM_006005 | NM_011716 | BU504246 |
| PDE6B | 04p16.3 | NM_000283 | NM_008806 | BU506473 |
| LRAT | 04q31.2 | NM_004744 | NM_023624 | BB284044 |
| PDE6A | 05q31.2-q34 | NM_000440 | NM_008805 | BQ946053 |
| GUCA1A | 06p21.1 | NM_000409 | NM_008189 | BM937552 |
| RDS(RP7) | 06p21.2-cen | NM_000322 | NM_008938 | BQ946742 |
| TULP1(RP14) | 06p21.3 | NM_003322 | BC014820 | BU506101 |
| ELOVL4 | 06q14 | NM_022726 | AF277093 | BU504885 |
| PEX1 | 07q21-q22 | NM_000466 | AK017309 | BG342406 |
| IMPDH1(RP10) | 07q31.1 | NM_000883 | NM_011829 | BI872626 |
| OPN1SW | 07q31.3-q32 | NM_001708 | NM_007538 | BU505452 |
| RP1 | 08q11-q13 | NM_006269 | AF146593 | BU504349 |
| TTPA | 08q13.1-q13.3 | NM_000370 | AK004882 | BB284649 |
| CNGB3 | 08q21-q22 | NM_019098 | NM_013927 | BM938767 |
| PHYH | 10p15.3-p12.2 | NM_006214 | NM_010726 | BI733397 |
| PCDH15 | 10q21-q22 | NM_033056 | NM_023115 | BF463579 |
| RGR | 10q23 | NM_002921 | NM_021340 | BM932762 |
| OAT | 10q26 | NM_000274 | NM_01697_ | BU505562 |
| ROM1 | 11q13 | NM_000327 | NM_009073 | BQ938664 |
| MYO7A | 11q13.5 | NM_000260 | NM_008663 | BU506306 |
| RDH5 | 12q13-q14 | NM_002905 | BC021372 | BI734215 |
| RB1 | 13q14.2 | NM_000321 | NM_009029 | BF466536 |
| RHOK | 13q34 | NM_002929 | NM_011881 | BI730057 |
| NRL(RP27) | 14q11 | NM_006177 | NM_008736 | BU505660 |
| RPGRIP1 | 14q11 | NM_020366 | NM_023879 | BU503646 |
| BBS4 | 15q22.3-q23 | NM_033028 | NM_033028 | BU506010 |
| NR2E3 | 15q23 | NM_014249 | NM_013708 | BU506000 |
| RLBP1 | 15q26 | NM_000326 | NM_020599 | BI872669 |
| CLN3 | 16p12.1 | NM_000086 | NM_009907 | BI732236 |
| CNGB1 | 16q13-q21 | NM_001297 | NM_001297 | BI736222 |
| BBS2 | 16q21 | NM_031885 | AF342737 | BM940513 |
| AIPL1(LCA4) | 17p13.1 | NM_014336 | AF296412 | BU505146 |
| GUCY2D | 17p13.1 | NM_000180 | NM_008192 | BI738500 |
| RP13 | 17p13.3 | NM_006445 | AB047391 | BQ946316 |
| UNC119 | 17q11.2 | NM_005148 | NM_011676 | BU506090 |
| PDE6G | 17q21.1 | NM_002602 | NM_012065 | BQ938436 |
| FSCN2 | 17q25 | NM_012418 | NM_012418 | BB279997 |
| OPA3 | 19q13.2-q13.3 | NM_025136 | NM_025136 | BF465192 |
| CRX(CORD2) | 19q13.3 | NM_000554 | NM_007770 | BU503524 |
| RP11 | 19q13.4 | NM_015629 | NM_027328 | BQ960012 |
| JAG1 | 20p12 | NM_000214 | NM_013822 | BM939721 |
| MKKS(BBS6) | 20p12 | NM_018848 | NM_021527 | BQ960058 |
| TIMP3 | 22q12.1-q13.2 | NM_000362 | NM_011595 | BE985781 |
| RP9(PIM1K) | 7p15.1-p13 | AX016710 | NM_018739 | BB283878 |
| CACNA1F | Xp11.23 | NM_005183 | NM_019582 | BU506115 |
| NDP | Xp11.3 | NM_000266 | NM_010883 | BG293757 |
| RP2 | Xp11.3 | NM_006915 | AJ303371 | BI736275 |
| NYX | Xp11.4 | NM_022567 | NM_022567 | BI732501 |
| RPGR(RP3) | Xp21.1 | NM_000328 | NM_011285 | BG295591 |
| DMD | Xp21.2 | NM_000109 | M68859 | BI729851 |
| RS1 | Xp22.2 | NM_000330 | AF084561 | BU504650 |
| PGK1 | Xq13.3 | NM_000291 | NM_008828 | BI871783 |
| CHM | Xq21.1-q21.3 | NM_000390 | NM_018818 | BI873103 |
| TIMM8A | Xq22 | NM_004085 | NM_013898 | BE988734 |
| OPN1MW | Xq28 | NM_000513 | NM_008106 | BU503734 |
| OPN1LW | Xq28 | NM_020061 | BC014826 | BU503734 |

8. Known photoreceptor gene:

| **Name** | **Chromosome** | **Human Ref** | **Mouse Ref** | **Core RTCs** |
| --- | --- | --- | --- | --- |
| GNAT2 | 01p13.1 | NM_005272 | NM_008141 | BU505641 |
| ABCA4 | 01p21-p22 | NM_000350 | NM_007378 | BU504330 |
| RPE65 | 01p31 | NM_000329 | AF410461 | BM932603 |
| CRB1(RP12) | 01q31-q32.1 | NM_012076 | NM_133239 | BU506277 |
| CNGA3 | 02q11 | NM_001298 | AJ243933 | BI730059 |
| SAG | 02q37.1 | NM_000541 | NM_009118 | BQ921528 |
| GNAT1 | 03p22 | NM_000172 | NM_008140 | BU505975 |
| RHO (RP4) | 03q21-q24 | NM_000539 | BC013125 | BU506109 |
| PROML1 | 04p | NM_006017 | NM_008935 | BU505217 |
| CNGA1 | 04p12-cen | NM_000087 | U19717 | BQ946527 |
| PDE6B | 04p16.3 | NM_000283 | NM_008806 | BU506473 |
| PDE6A | 05q31.2-q34 | NM_000440 | NM_008805 | BQ946053 |
| GUCA1A | 06p21.1 | NM_000409 | NM_008189 | BM937552 |
| RDS(RP7) | 06p21.2-cen | NM_000322 | NM_008938 | BQ946742 |
| TULP1(RP14) | 06p21.3 | NM_003322 | BC014820 | BU506101 |
| ELOVL4 | 06q14 | NM_022726 | AF277093 | BU504885 |
| PEX1 | 07q21-q22 | NM_000466 | AK017309 | BG342406 |
| IMPDH1(RP10) | 07q31.1 | NM_000883 | NM_011829 | BI872626 |
| OPN1SW | 07q31.3-q32 | NM_001708 | NM_007538 | BU505452 |
| RP1 | 08q11-q13 | NM_006269 | AF146593 | BU504349 |
| CNGB3 | 08q21-q22 | NM_019098 | NM_013927 | BM938767 |
| PCDH15 | 10q21-q22 | NM_033056 | NM_023115 | BF463579 |
| RGR | 10q23 | NM_002921 | NM_021340 | BM932762 |
| ROM1 | 11q13 | NM_000327 | NM_009073 | BQ938664 |
| MYO7A | 11q13.5 | NM_000260 | NM_008663 | BU506306 |
| RDH5 | 12q13-q14 | NM_002905 | BC021372 | BI734215 |
| RHOK | 13q34 | NM_002929 | NM_011881 | BI730057 |
| NRL(RP27) | 14q11 | NM_006177 | NM_008736 | BU505660 |
| RPGRIP1 | 14q11 | NM_020366 | NM_023879 | BU503646 |
| NR2E3 | 15q23 | NM_014249 | NM_013708 | BU506000 |
| RLBP1 | 15q26 | NM_000326 | NM_020599 | BI872669 |
| CNGB1 | 16q13-q21 | NM_001297 | NM_001297 | BI736222 |
| BBS2 | 16q21 | NM_031885 | AF342737 | BM940513 |
| AIPL1(LCA4) | 17p13.1 | NM_014336 | AF296412 | BU505146 |
| GUCY2D | 17p13.1 | NM_000180 | NM_008192 | BI738500 |
| UNC119 | 17q11.2 | NM_005148 | NM_011676 | BU506090 |
| PDE6G | 17q21.1 | NM_002602 | NM_012065 | BQ938436 |
| FSCN2 | 17q25 | NM_012418 | NM_012418 | BB279997 |
| CRX(CORD2) | 19q13.3 | NM_000554 | NM_007770 | BU503524 |
| JAG1 | 20p12 | NM_000214 | NM_013822 | BM939721 |
| CACNA1F | Xp11.23 | NM_005183 | NM_019582 | BU506115 |
| RP2 | Xp11.3 | NM_006915 | AJ303371 | BI736275 |
| RPGR(RP3) | Xp21.1 | NM_000328 | NM_011285 | BG295591 |
| RS1 | Xp22.2 | NM_000330 | AF084561 | BU504650 |
| CHM | Xq21.1-q21.3 | NM_000390 | NM_018818 | BI873103 |
| OPN1MW | Xq28 | NM_000513 | NM_008106 | BU503734 |
| OPN1LW | Xq28 | NM_020061 | BC014826 | BU503734 |

9. Count genes in cytobands:

9.1 download Ensembl gene record from: "http://genome.ucsc.edu/goldenPath/28jun2002/database/ensGene.txt.gz"

9.2 download cytoband definition from: "http://genome.ucsc.edu/goldenPath/28jun2002/database/cytoBand.txt.gz"

9.3 for each cytoband, count howmany Ensembl genes located in this region.

9.4 programs :

count_cytoband_genes.py : work as described at 9.3
