# Supplementary figures and images for: Comprehensive in silico functional specification of mouse retina transcripts
Source: BMC Genomics. 2005 Mar 18;6:40. doi: 10.1186/1471-2164-6-40 (PMC1083414; doi:10.1186/1471-2164-6-40)

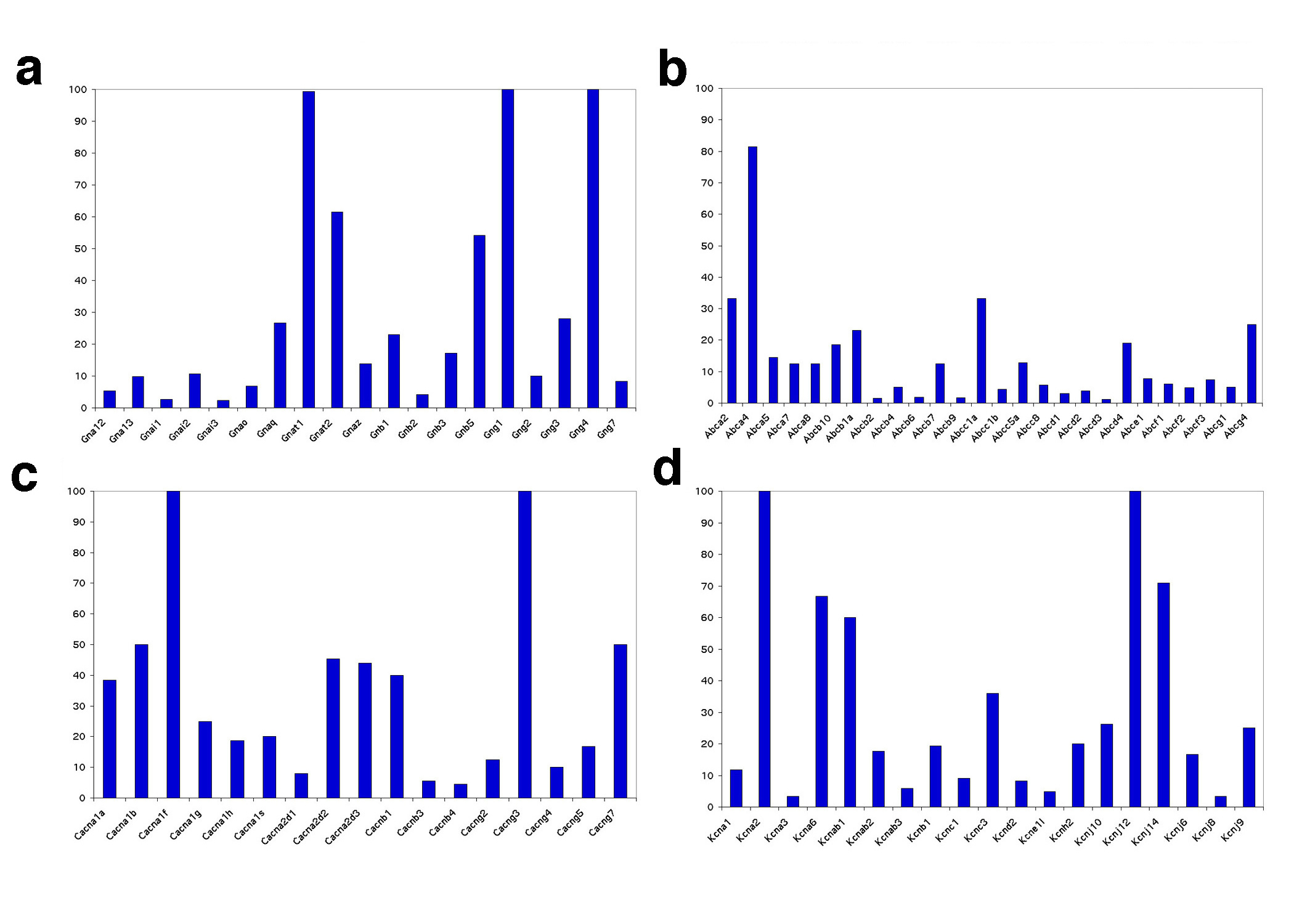

Supplement: Additional File 6 — Samples of the percentage of RTCs in different gene families. a, Heterotrimeric guanine nucleotide-biding proteins. b, ATP-binding cassette (ABC) transporter superfamily. c, Voltage-dependent calcium channel proteins. d, Voltage-gated potassium channel proteins. [file 1471-2164-6-40-S6.jpeg]

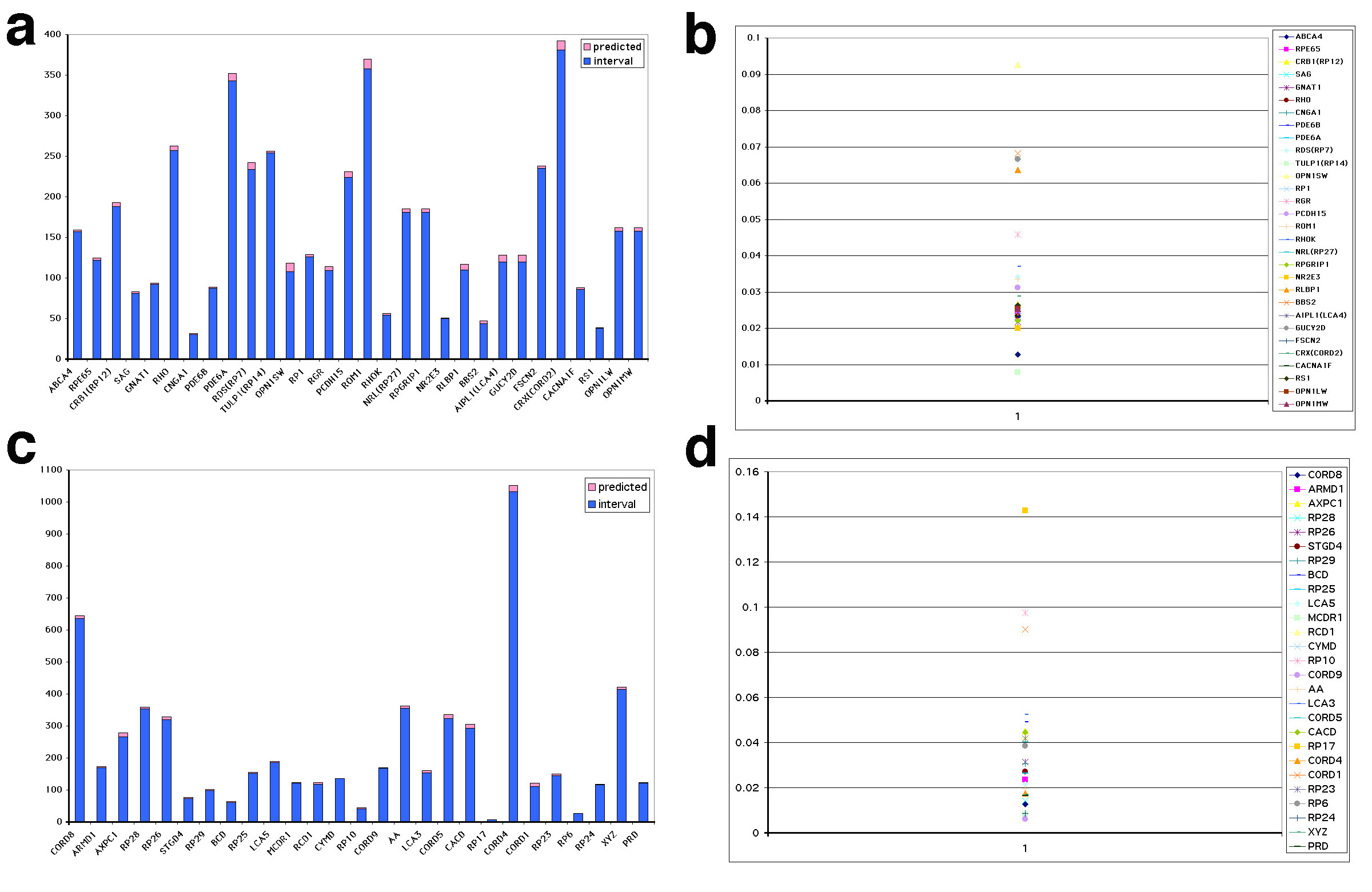

Supplement: Additional File 7 — Candidate genes for human retina disease loci. a, Gene numbers of chromosome interval and concentrated retina enriched gene pool in human known retina disease gene loci. b, Concentrated ratio between retina enriched gene pool and whole interval genes of the loci for human known retina disease gene. c, Gene numbers of chromosome interval and concentrated retina enriched gene pool in some human known retina disease loci. d, Concentrated ratio between retina enriched gene pool and whole interval genes of the loci for human retina disease. [file 1471-2164-6-40-S7.jpeg]
