# Supplementary material for: Comprehensive in silico functional specification of mouse retina transcripts
Source: BMC Genomics. 2005 Mar 18;6:40. doi: 10.1186/1471-2164-6-40 (PMC1083414; doi:10.1186/1471-2164-6-40)
Supplement: Additional File 10 — Additional methods. [file 1471-2164-6-40-S10.doc]

Additional Methods:

Protocol for microarray experiments using MR.Chips 9.2k

A. Retina dissection

Retina is dissected from eyeball without contamination with lens, iris, cornea, and cililary body. Average 10 to 20 retinas and other organs are pooled for RNA isolation.

B. RNA isolation from mouse retina by TRIzol

1. 1ml TRIzol
2. 50-100mg Tissues from retina
3. Homogenize (glass, Telfen) well
4. Spin at 12,000 x g for 10 min at 4 ˚C, if there are insoluble stuff
5. transfer to new tube
6. stay room tempreture for 5 min
7. Add 0.2 ml of Chloroform per ml TRLzol
8. Cap securely, shack gentally for 15 sec
9. Stay room tempreturefor 5 min
10. Spin at 12,000 x g for 15 min at 4 ˚C
11. Transfer the aqueous phase to a fresh tube
12. Preciptate RNA by mixing with 0.5 ml Isopropyl Alcohol
13. Stay at room tempreture for 10 min
14. Spin 12,000 x g for 10 min at 4 ˚C
15. Remove the supernate
16. Wash with 1 ml 75% ethanol
17. Mix by vortex
18. Spin 7,500 x g for 5 min at 4 ˚C
19. Dry pellet on air for 5-10 min
20. Dissolve pellet in RNAase-free water (100 ul, if for clean up)
21. Incubate at 55 to 60 ˚C for 10 min
22. Stored at –80 ˚C or clean up

# C. RNA clean up by RNeasy Mini protocol

1. Adjust sample to a volume of 100 ul with RNase-free water.
2. Add 350 ul Buffer RLT (βME ready) to the sample and mix thoroughly.
3. Add 250 ul ethanal (100%) to the lysate and mix well, do not centrifuge.
4. Apply 700 ul to a RNeasy Mini Spin Colum sitting in a Collection Tube.
5. Centrifuge for 15 sec at 10,000 rpm.
6. Transfer the RNeasy Colum into a new 2 ml Collection Tube (supplied).
7. Add 500 ul Buffer RPE.
8. Centrifuge for 15 sec at 10,000 to wash.
9. Discard flow through and reuse the Collection Tube.
10. Pipet 500 ul Buffer RPE onto the colum.
11. Centrifuge for 2 min at maximum speed to dry.
12. Transfer the RNeasy Colum into a new 1.5 ml collection tube.
13. Pipet 30-50 ul of RNase-free water directly onto the RNeasy Membrane.
14. Centrifuge for 1 min at 10,000 rpm to elute.
15. Repeat if the expected RNA yield is > 30ug.

D. RNA Concentration by Microcon (Millipore)

1. Insert Microcon sample reservoir into vial.
2. Pipette RNAas free H2O into sample reservoir (0.5 ml maximum volume) without touching the membrane with the pipette tip.
3. Seal with attached cap.
4. Place assembly in a compatible centrifuge.
5. Spin at 14,000 rpe for 12 min. for Clear tube, 500ul samples reduced to 10ul.
6. Separate vial from sample reservoir.
7. Place sample reservoir upside down in a new vial.
8. Spin 3 min. at 1000 x g (or pulse briefly) to transfer concentrate to vial.
9. Separate sample reservoir and seal cap.

# E. Genisphere 2-Step Array Detection Protocol

*Reagents:*

Genisphere Kit

RNase-free water

Tris-HCl, pH 7.5

0.5M NaOH/50mM EDTA

TE pH 8.0

5M NaCl

100% Ethanol

0.5ml clean tube

*Reaction mix:*

10 ul RNA (5 ug each in RNase-free water)

1.5 ul Cy3 or Cy5 RT primer (5 pmol/ul) (Vial 2)

11.5 ul

• Mix, place tubes at 80°C for 10 min.

• Snap cool on ice for 2 min.

Add:

4 ul 5X RT buffer (Vial 5)

1 ul dNTPs (Vial 4)

1 ul Superasin (Vial 12)

1 ul RT Enzyme (Vial 3)

3 ul RNase-free water

20 ul Final Reaction Volume 9.5 ul each sample

• Mix gently, spin contents to bottom of tube.

• Incubate at 42°C for 90 min.

• Stop reaction with 3.5 ul 0.5M NaOH/50mM EDTA, incubate at 65°C for 10 min.

• Neutralize with 5 ul 1M Tris-HCl, pH 7.5.

• Combine Cy3 and Cy5 reactions. Rinse empty tube with 42 ul TE pH 8.0 and add to combined fractions.

• Add 4 ul 5M NaCl, mix.

• Add 3 ul linear acrylamide co-precipitant (Vial 11).

• Add 400 ul 100% Ethanol and vortex well. Place sample at-80°C for 30 min. Vortex well and stay at room temperature for min. Spin sample at 4°C in centrifuge for 15 min.

• Aspirate Ethanol with a pipette. Briefly rinse pellet with ~15 ul 100% Ethanol, and remove with pipette. Then 75% Ethanol, and remove with pipette. Dry pellet at 65°C for 10 min.

*Slide Denature:*

• Make solution with 50% Formamide and 3x SSC

100% Formamide 10 ml

20x SSC 3 ml

H2O 7 ml

Total 20 ml

• Total 25 ul onto one array.

• Cover slip.

• Incubate on heat blocker-metal for 2 min. at 76°C.

• Transfer slide into a chamber. Chamber buffer = hybridization buffer 55ul.

• Incubate under water-bath at 50°C for at least 30 min.

• Wash by RNase-free water.

• Go through 70%, 100% ethanal quickly.

• Air dry at dark.

*Hybridization:*

First day

• Assemble 1st step hybridization mix as follows:

Dry pellet

12.0 ul vial 7 hybridization buffer

2.0 ul LNA blocker

10.0 ul RNase Free water

24 ul Total Reaction Volume

• Vortex and place at 80°C for 10 min. Vortex well, and spin contents to bottom of tube.

• Pipette hybridization mixture onto array.

• Cover slip and hybridize overnight at 50°C for 9 hr.

• Wash arrays in the dark as follows:

2X SSC, 0.2% SDS for 15 min at 55°C.

2X SSC for 10 min at room temperature.

0.2X SSC for 10 min at room temperature.

• Place slides in 50 ml centrifuge tubes and spin at 1,000 rpm for 3 min.

Second day

• Assemble 2nd step hybridization mix as follows:

2.0 ul Cy3 capture reagent

2.0 ul Cy5 capture reagent

1.0 ul High End Differential Enhancer (cDNA kit only)

12.0 ul Vial 7 hybridization buffer

7.0 ul RNase Free water

24 ul Total Reaction Volume

• Vortex and heat mixture at 80°C for 10 min.

Spin contents to the bottom of the tube.

(Note: Be sure to thoroughly thaw and vortex capture reagents before use.

Heat at 55°C for 10 min if needed.)

• Apply hybridization mixture to array

• Cover slip and hybridize at 50°C for at for 9 hours.

• Wash and spin arrays as above.

2X SSC, 0.2% SDS for 15 min at 60°C.

2X SSC for 10 min from 60°C to room temperature.

0.2X SSC for 5 min at room temperature x 2 times.

• Place slides in 50 ml centrifuge tubes and spin at 1,000 rpm for 3 min.

• Store arrays in the dark until ready to scan.

F. Imaging and data collection

Slides were scanned on a GenePix 4000B scanner (Axon Instruments) under same condition and the data were manipulated with GenePix software Version 4.0 (Axon Instruments). Before statistic analysis all primary data, the intensities are normalized by median volume for each channel.

G. Statistics methods for microarray data

Three or more repeats of each microarray data are analyzed by following methods. To determine the retina enriched genes and mark the group of these genes which expressed highly in late stages compared with the early stages of development, we use two kinds of filters. First, we used fold-change filter. We deemed the genes which expressed 2-fold (log2>1) higher in PN21 retina than in PN1 retina, and 2-fold higher in PN35 wild-type retina than in P35 *Rd1* retina as well, as the retina enriched genes which expressed highly in adulthood. The genes which expressed 2-fold higher in retina compared with both body and brain respecitively were defined as retina enriched genes. Since the genes with 2-3 folds changes carried the most variance, we used a more conservative filter: we first mark the genes with greater than 3-fold increase in PN21, then enrolled the rest of genes which expressed higher in PN21 than in PN1 retina in student t-test. the genes with p<0.05 were added in the target genes list. Same filter was used in substrating yielding the retina enriched genes from the Wild-type/*Rd1*, retina/body and retina/brain comparison.

Statistic calculations are done by R 1.8.1 or R 1.7.1 ([http://cran.us.r-project.org](http://cran.us.r-project.org/)) or Microsoft Excel 2000 (Microsoft).
